# Neck Circumference Is Associated with Muscle Sympathetic Nerve Activity in Overweight and Obese Men but Not Women

**DOI:** 10.3389/fphys.2017.00203

**Published:** 2017-04-06

**Authors:** Nora E. Straznicky, Mariee T. Grima, Carolina I. Sari, Nina Eikelis, Paul J. Nestel, John B. Dixon, Gavin W. Lambert, Markus P. Schlaich, Sarah E. Phillips, Elisabeth A. Lambert

**Affiliations:** ^1^Human Neurotransmitters Laboratory, Baker Heart and Diabetes InstituteMelbourne, VIC, Australia; ^2^Cardiovascular Nutrition Laboratory, Baker Heart and Diabetes InstituteMelbourne, VIC, Australia; ^3^Department of Primary Health Care, Monash UniversityMelbourne, VIC, Australia; ^4^Faculty of Medicine, Nursing and Health Sciences, Monash UniversityMelbourne, VIC, Australia; ^5^Royal Perth Hospital Unit, School of Medicine and Pharmacology, University of Western AustraliaCrawley, WA, Australia; ^6^Faculty of Health, Arts and Design, Iverson Health Innovation Research Institute, Swinburne University of TechnologyMelbourne, VIC, Australia

**Keywords:** neck circumference, sympathetic nervous system activity, obesity, gender differences, metabolic syndrome, cardiovascular risk, insulin resistance

## Abstract

**Background:** Neck circumference (NC) is a predictor of cardiometabolic risk. The objective of this study was to explore the relationship of NC to muscle sympathetic nerve activity (MSNA) within an overweight and obese population.

**Methods:** The study design was a retrospective cross-sectional analysis. Un-medicated persons (72 men, 53 postmenopausal women) aged 56 ± 1 years (mean ± SEM) with body mass index (BMI) 32.8 ± 0.4 kg/m^2^, were studied. NC was measured together with traditional anthropometric measures, supine blood pressure, fasting blood lipids, insulin, and glucose. Insulin sensitivity was assessed by homeostasis model (HOMA-IR) and Matsuda Insulin Sensitivity Index (ISI) derived from 75-g oral glucose tolerance test. Resting multiunit MSNA was recorded by microneurography in the peroneal nerve and expressed as burst frequency and burst incidence.

**Results:** Men within the highest tertile of NC had significantly higher fasting and post-glucose plasma insulin levels (insulin AUC_0−120_), HOMA-IR, non-esterified fatty acids, MSNA (45 ± 2 vs. 36 ± 2 bursts per min; 69 ± 3 vs. 58 ± 3 bursts per 100 hb) and heart rate, and lower Matsuda ISI compared to men in the lowest tertile (*P* all <0.05). In stepwise regression analyses, NC alone explained 12%, and together with insulin AUC_0−120_ it accounted for 22%, of the variance in MSNA in men. In women, NC was associated with anthropometric measures but not with MSNA or metabolic indices.

**Conclusions:** Among overweight and obese men, NC was independently associated with elevated MSNA and hyperinsulinemia, and thus may be relevant to cardiometabolic risk prediction. The biological basis of gender differences merits further elucidation.

## Introduction

Excess upper-body adiposity, particularly in the visceral region, confers increased risk of developing metabolic syndrome, type 2 diabetes and cardiovascular disease (Neeland et al., 2012; Abraham et al., 2015). Since neck fat compartments expand with increasing adiposity, interest has focused on the utility of neck circumference (NC), a simple anthropometric measurement and proxy for upper-body subcutaneous fat, to predict cardiometabolic risk (Torriani et al., 2014). Imaging studies have established that neck adipose tissue quantified by computed tomography correlates positively with abdominal visceral fat and cardiovascular risk factors, and is predictive of metabolic syndrome and obstructive sleep apnea severity (Karimi et al., 2010; Li et al., 2014; Torriani et al., 2014). In large population studies, such as the Framingham Heart Study and the Brazilian Longitudinal Study of Adult Health (ELSA-Brasil), NC was positively associated with cardiovascular risk factors and subclinical atherosclerosis, even after adjustment for visceral adipose tissue and/or body mass index (BMI) (Preis et al., 2010; Baena et al., 2016). This suggests that fat accumulation in the neck may confer risk beyond visceral adipose tissue accumulation. Moreover, in longitudinal analyses, NC was predictive of incident type 2 diabetes in the Framingham cohort, and of future cardiovascular events and all-cause mortality in high-risk cardiology outpatients (Preis et al., 2013; Dai et al., 2016).

Elevated sympathetic nervous system (SNS) activity is a characteristic of obesity that relates to a number of pathogenic factors including central body fat distribution, hyperinsulinemia secondary to insulin resistance, baroreflex impairment, and the presence of obstructive sleep apnea (OSA) (Narkiewicz et al., 1998a; Beske et al., 2002; Grassi et al., 2004). Chronic sympathetic activation promotes unfavorable alterations in cardiovascular structure and function (e.g., arterial stiffening, cardiac enlargement, diastolic dysfunction, glomerular hyperfiltration) and decreased insulin sensitivity, via both blood pressure dependent and independent effects. Consequently, it is an important mediator of target organ damage and metabolic risk in both obese and hypertensive states (Masuo et al., 1997; Schlaich et al., 2015). To our knowledge, no study to date has examined the relationship of NC to SNS activity, which may be of relevance to cardiovascular risk stratification. Therefore, the aim of the present study was to perform gender-specific cross-sectional analyses to determine whether NC is independently associated with muscle sympathetic neural activity (MSNA) in overweight and obese subjects. The technique of microneurography was used to directly quantify efferent post-ganglionic sympathetic outflow directed to skeletal muscle vasculature in the lower leg.

## Methods

### Study population

Subjects participating in clinical trials of weight loss and pharmacological insulin-sensitization, during the period 2003–2014 (registered as NCT00163943, NCT00408850, and NCT 01771042), who had both NC and MSNA measurements at baseline, were included in the present analysis. Selection criteria and experimental methodologies were similar across all trials. Subjects were recruited from the general population via newspaper advertisements and telephone screening. Entry criteria were: non-smoker, aged 45–70 years, with a BMI >27 kg/m^2^ and weight stable (± 1 kg) in the previous 6 months. Females were postmenopausal. Exclusion criteria comprised history of cardiovascular, cerebrovascular, renal, liver, or thyroid diseases; use of continuous positive airway pressure treatment; and consumption of medications that could affect study parameters (e.g., oral hypoglycemics, cholesterol-lowering, anti-hypertensive, anti-depressant and hormone replacement therapies). All participants signed an informed, witnessed, consent form. Study protocols were approved by the Alfred Hospital Human Research Ethics Committee.

### Clinical investigations

Participants attended our clinical research unit at 8 am, having fasted overnight and abstained from caffeine for 18 h and alcohol and heavy exercise for 36 h. Body weight was measured in light indoor clothing and bare feet, using a digital scale. Waist and hip circumferences were measured at the midpoint between the lowest rib and the iliac crest and at the greater trochanter, respectively. NC was measured with a flexible plastic tape, with subjects standing upright and their head in the Frankfort horizontal plane. Measurements were made in the midway of the neck, just below the laryngeal prominence (Ben-Noun et al., 2001). Supine resting blood pressure was recorded as the average of 5 readings after 5 min rest using a Dinamap monitor (Model 1846SX, Critikon Inc, Tampa, FL, USA). Fasting venous blood was drawn for the measurement of lipid profile, glucose and insulin, biochemistry, liver function tests, non-esterified fatty acids (NEFA), leptin, high-sensitivity C-reactive protein (hs-CRP) and plasma renin activity (PRA). Presence of metabolic syndrome components were determined according to the harmonized definition, using waist circumference cut-points of ≥102 cm in men and ≥88 cm in women (Alberti et al., 2009).

Assessments of MSNA, cardiac baroreflex sensitivity (BRS) and oral glucose tolerance were performed together on the same morning, in the supine position, in a temperature controlled (22°C) research room. Subjects voided prior to commencement. A tungsten microelectrode (FHC, Bowdoinham, ME, USA) was inserted into the right peroneal nerve at the fibular head (Vallbo et al., 2004). A subcutaneous reference electrode was positioned 2 to 3 cm away from the recording site. Standard criteria were used to ascertain a MSNA site. The nerve signal was amplified (X50,000), filtered (bandpass, 700 to 2,000 Hz), and integrated. Intra-arterial blood pressure, ECG, respiration and MSNA were digitized with a sampling frequency of 1,000 Hz (PowerLab recording system, model ML 785/8SP, ADI Instruments). Resting measurements were recorded over a 15-min period and averaged. Sympathetic bursts were counted manually and expressed as burst frequency (bursts/min) and burst incidence (bursts/100 heart beats). Spontaneous cardiac BRS was assessed by the sequence method (Parati et al., 2004). The slope of the regression line between cardiac interval and systolic blood pressure was calculated for validated sequences of three or more consecutive heartbeats in which systolic blood pressure progressively increased and cardiac interval lengthened (type 1 sequence) or systolic blood pressure progressively decreased and cardiac interval shortened (type 2 sequence), with a lag of 1 beat. Recordings were averaged over a 15-min period of supine rest.

After completion of microneurography, subjects underwent a standard 75-g oral glucose tolerance test (OGTT, Glucaid, Fronine PTY, LTD, Taren Point, NSW 2229, Australia) with 30-min intravenous blood sampling for determination of glucose and insulin responses. Homeostasis model assessment of insulin resistance (HOMA-IR) and the Matsuda insulin sensitivity index (ISI) were calculated as indices of insulin resistance and sensitivity, respectively (Matthews et al., 1985; Matsuda and DeFronzo, 1999). The insulinogenic index (Δ insulin 0–30 min divided by Δ glucose 0–30 min) was used to assess pancreatic β-cell function.

### Laboratory measurements

Plasma glucose and lipid profile were measured on a commercial analytical system (Architect C18000 analyzer, Abbott Laboratories, Illinois, USA). H*s*-CRP was quantified by immunoturbidimetric assay, insulin, leptin and PRA by radio-immuno assay (Linco Research, Inc, Missouri, USA; REN-CT2, CIS bio international, France), and NEFA by enzymatic colorimetry (Wako Pure Chemical Industries, Ltd, Osaka, Japan).

### Statistical analysis

Statistical analysis was performed using SigmaStat Version 3.5 (Systat Software Inc, Point Richmond, CA, USA). Data are presented as the mean ± SEM for normally distributed data and as the median [interquartile range] for nonparametric data. Data from men and women were separately stratified into tertiles of NC. Comparisons of MSNA, anthropometric, metabolic and cardiovascular parameters across tertiles were made by one-way ANOVA and Kruskal-Wallis one-way ANOVA on ranks, as appropriate. The Holm-Sidak test was used to adjust for multiple comparisons. Univariate associations between MSNA and other variables were assessed using Pearson's correlations. Non-parametric data were log-transformed. Forward stepwise linear regression analysis, was performed to determine whether NC was independently associated with MSNA within each gender. Statistical significance was accepted at the *P* < 0.05 (two-tailed) level.

## Results

Of the 125 subjects studied, 72 were men and 53 were women. The two genders had similar BMI, HOMA-IR, Matsuda ISI, glucose tolerance, MSNA and cardiac BRS. However, the women were significantly older (58 ± 1 vs. 54 ± 1 years), with higher plasma HDL- and LDL-cholesterol, leptin and NEFA concentrations, and lower alanine aminotransferase (ALT) and gamma-glutamyltransferase (GGT) levels, body weight, waist circumference, waist to hip ratio and NC compared to the men. (*P* all < 0.01).

**Figure 1:**
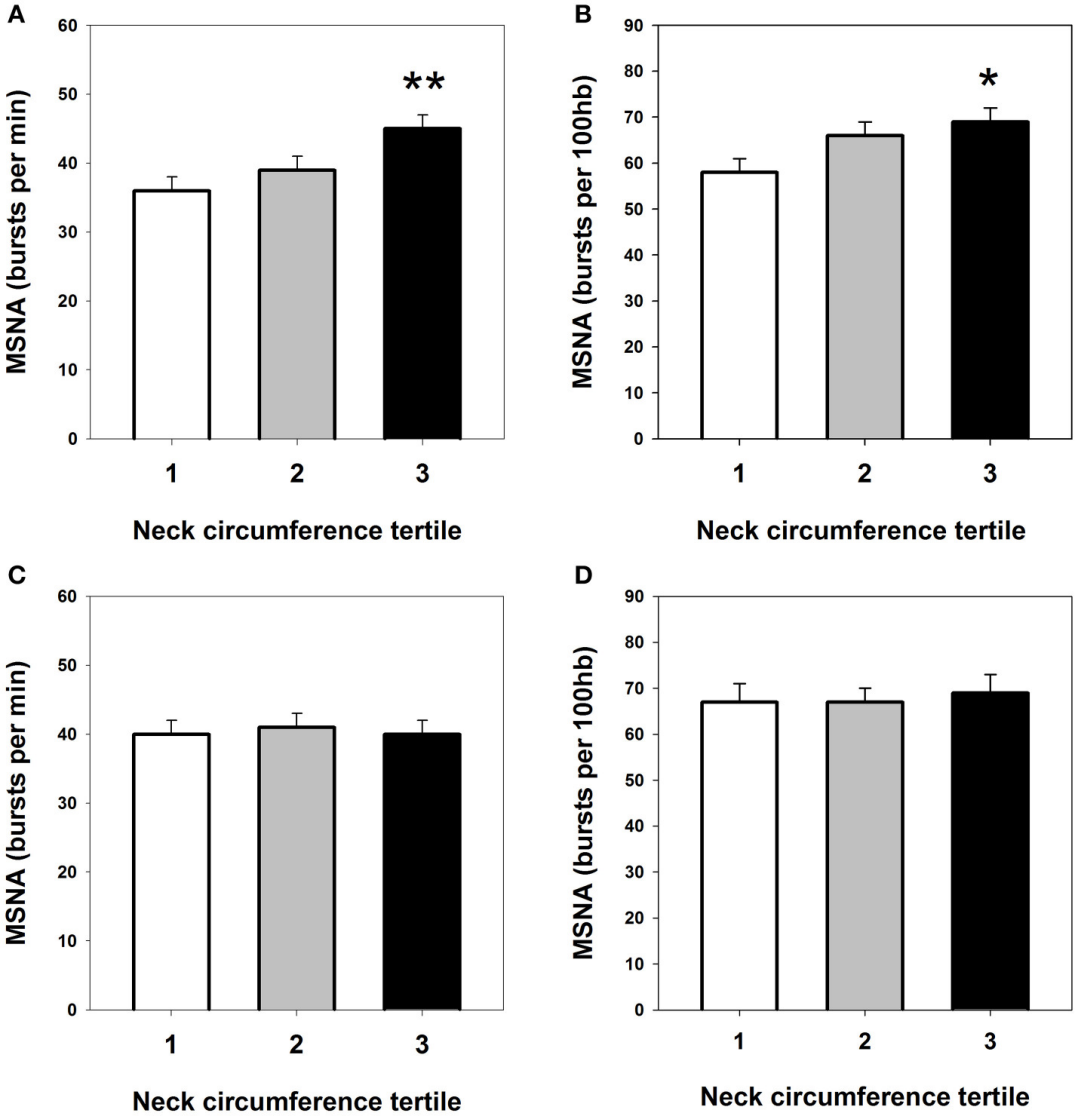
**Microneurographic recordings of resting muscle sympathetic nerve activity (MSNA), expressed as burst frequency and burst incidence, within tertiles of neck circumference in men (upper panels; A,B) and women (lower panels; C,D)**. ^*^P < 0.05 and ^**^P < 0.01 vs. tertile 1.

Tables 1, 2 summarize clinical and demographic data according to NC tertiles in men and women, respectively. Men within the highest tertile of NC had significantly higher anthropometric indices, fasting and post-glucose plasma insulin levels (insulin area under the curve, AUC_0−120_), HOMA-IR, NEFA, and lower Matsuda ISI compared to men in the lowest tertile (*P* all < 0.05). Blood pressure and cardiac BRS did not differ across NC tertiles. In women, BMI, waist circumference and waist to hip ratio increased significantly across NC groups, whereas metabolic and cardiovascular parameters did not differ between tertiles. Figure 1 shows that men within the highest NC tertile had significantly higher MSNA burst frequency (45 ± 2 vs. 36 ± 2 bursts/min) and burst incidence (69 ± 3 vs. 58 ± 3 bursts/100 hb) compared to the lowest NC tertile. In women MSNA did not differ between NC tertiles (lower panels, Figure 1).

**Table 1:** **Demographic and clinical data in relation to neck circumference tertile in men**.

**Variable**	**Tertile 1 (*n* = 24)**	**Tertile 2 (*n* = 24)**	**Tertile 3 (*n* = 24)**	***P***
Neck circumference (cm)	40.5 ± 0.3	43.4 ± 0.1^***^	46.9 ± 0.5^***^^‡^	<0.001
Age (yrs)	54 ± 1	56 ± 1	53 ± 1	0.18
Weight (kg)	91.9 ± 1.7	101.2 ± 2.3^**^	118.6 ± 3.0^***^^‡^	<0.001
BMI (kg/m^2^)	29.9 ± 0.4	32.5 ± 0.5^**^	36.7 ± 0.9^***^^‡^	<0.001
Waist circumference (cm)	104.6 ± 1.2	111.2 ± 1.6^**^	121.9 ± 2.1^***^^‡^	<0.001
Waist: Hip ratio	0.97 ± 0.01	0.98 ± 0.01	0.99 ± 0.01	0.13
Leptin (ng/ml)	6.3 (5.2, 10.1)	7.1 (6.1, 11.9)	9.6 (6.7, 19.5)	0.09
LDL-cholesterol (mmol/L)	3.6 ± 0.2	3.1 ± 0.1	3.3 ± 0.2	0.08
HDL-cholesterol (mmol/L)	1.00 (0.90, 1.15)	1.05 (1.00, 1.20)	1.10 (0.90, 1.20)	0.88
Triglycerides (mmol/L)	1.4 (0.9, 2.1)	1.4, (1.2, 2.1)	1.8 (1.3, 2.6)	0.18
NEFA (mEq/L)	0.43 ± 0.03	0.40 ± 0.03	0.51 ± 0.03^*^^‡^	0.02
Hs-CRP (mg/L)	1.7 (1.2, 4.9)	1.7 (1.3, 2.7)	2.9 (2.1, 4.7)	0.11
ALT (mU/L)	25 (21, 30)	28 (22, 45)	30 (26, 40)	0.17
GGT (mU/L)	34 (29, 45)	33 (23, 58)	31 (25, 48)	0.66
Fasting glucose (mmol/L)	5.6 ± 0.1	6.0 ± 0.1^*^	5.8 ± 0.1	0.048
Fasting insulin (mU/L)	13.4 ± 1.1	16.4 ± 1.4	19.9 ± 1.4^***^	0.003
HOMA-IR	3.37 ± 0.28	4.48 ± 0.41	5.14 ± 0.36^***^	0.003
2-h glucose (mmol/L)	9.1 ± 0.5	10.1 ± 0.6	9.8 ± 0.4	0.31
Insulin AUC_0−120_ (mU min L^−1^)	8608 ± 619	9112 ± 842	11903 ± 1169^*^	0.03
Matsuda ISI	2.54 (2.20, 3.35)	2.09 (1.70, 3.09)	1.87 (1.41, 2.22)^***^	0.001
Insulinogenic index (mU insulin mmol^−1^ glucose)	14.9 (9.4, 23.1)	15.3 (9.3, 21.6)	17.8 (12.4, 29.9)	0.22
Systolic BP (mmHg)	132 ± 3	133 ± 3	130 ± 3	0.75
Diastolic BP (mmHg)	78 ± 2	78 ± 2	76 ± 2	0.69
Heart rate (bpm)	62 ± 2	59 ± 2	65 ± 2^†^	0.04
Cardiac BRS (msec/mmHg)	10.8 (6.7, 17.0)	13.7 (9.2, 16.0)	10.8 (7.7, 15.0)	0.44
PRA (ng/ml/h)	0.69 (0.32, 0.99)	0.76 (0.39, 1.22)	0.73 (0.32, 1.06)	0.86
MetS components (n)	2.5 (2.0, 3.0)	3.0 (2, 3.5)	3.0 (2.0, 4.0)	0.31

*Values are mean ± SEM or median (IQ range). ALT, alanine aminotransferase; BP, blood pressure; BRS, baroreflex sensitivity; GGT, gamma-glutamyltransferase; HDL, high density lipoprotein; HOMA-IR, homeostasis model assessment-insulin resistance; hs-CRP, high-sensitivity C-reactive protein; ISI, insulin sensitivity index; LDL, low-density lipoprotein; MetS, metabolic syndrome, NEFA, non-esterified fatty acids; PRA, plasma renin activity*.

* *P < 0.05*,

** P < 0.01 and

*** P < 0.001 vs. tertile 1;

† *P < 0.05*,

‡ *P < 0.01 vs. tertile 2*.

**Table 2:** **Demographic and clinical data in relation to neck circumference tertile in women**.

**Variable**	**Tertile 1 (*n* = 18)**	**Tertile 2 (*n* = 18)**	**Tertile 3 (*n* = 17)**	***P***
Neck circumference (cm)	34.8 ± 0.3	37.1 ± 0.2^***^	40.6 ± 0.4^***^^‡^	<0.001
Age (yrs)	59 ± 1	57 ± 1	59 ± 1	0.61
Weight (kg)	77.8 ± 2.2	87.9 ± 2.1^**^	96.3 ± 2.9^***^^†^	<0.001
BMI (kg/m^2^)	30.5 ± 0.6	32.1 ± 0.6	35.1 ± 1.0^***^^‡^	<0.001
Waist circumference (cm)	93.4 ± 1.4	99.2 ± 1.4^*^	108.9 ± 2.5^***^^‡^	<0.001
Waist: Hip ratio	0.82 ± 0.01	0.85 ± 0.01	0.89 ± 0.01^***^^‡^	<0.001
Leptin (ng/ml)	21.6 (17.7, 24.6)	23.2 (15.4, 41.9)	25.0 (16.8, 31.0)	0.84
LDL-cholesterol (mmol/L)	4.1 ± 0.2	3.5 ± 0.2	3.6 ± 0.2	0.13
HDL-cholesterol (mmol/L)	1.35 (1.30, 1.60)	1.30 (1.20, 1.40)	1.30 (1.20, 1.43)	0.30
Triglycerides (mmol/L)	1.6 (1.4, 1.8)	1.2 (1.1, 1.6)	1.4 (1.0, 2.0)	0.22
NEFA (mEq/L)	0.59 ± 0.05	0.61 ± 0.04	0.63 ± 0.05	0.88
Hs-CRP (mg/L)	2.4 (1.0, 4.3)	2.4 (1.2, 3.3)	3.0 (1.7, 4.8)	0.56
ALT (mU/L)	19 (17, 25)	19 (15, 26)	25 (21,34)	0.08
GGT (mU/L)	22 (20, 38)	19 (16, 27)	22 (16, 31)	0.40
Fasting glucose (mmol/L)	5.7 ± 0.1	5.7 ± 0.1	5.6 ± 0.1	0.95
Fasting insulin (mU/L)	17.0 ± 1.1	17.4 ± 1.7	17.4 ± 1.1	0.97
HOMA-IR	4.29 ± 0.28	4.43 ± 0.43	4.44 ± 0.33	0.95
2-h glucose (mmol/L)	9.6 ± 0.4	9.9 ± 0.7	9.7 ± 0.6	0.94
Insulin AUC_0−120_ (mU min L^−1^)	10,829 ± 1044	10,660 ± 827	11,668 ± 1008	0.75
Matsuda ISI	2.03 (1.81, 2.69)	2.11 (1.89, 2.56)	1.99 (1.49, 2.48)	0.88
Insulinogenic index (mU insulin mmol^−1^ glucose)	15.6 ± 2.0	16.5 ± 2.6	16.5 ± 1.8	0.95
Systolic BP (mmHg)	127 ± 4	125 ± 4	126 ± 4	0.98
Diastolic BP (mmHg)	72 ± 2	69 ± 2	69 ± 1	0.51
Heart rate (bpm)	64 ± 1	63 ± 2	61 ± 2	0.58
Cardiac BRS (msec/mmHg)	13.3 ± 1.7	16.1 ± 2.2	12.8 ± 1.3	0.35
PRA (ng/ml/h)	0.60 ± 0.09	0.61 ± 0.14	0.78 ± 0.13	0.53
MetS components (n)	3.0 (2.0, 3.0)	2.0 (2.0, 3.0)	3.0 (2.0, 4.0)	0.74

*Values are mean ± SEM or median (IQ range)*.

* *P < 0.05*,

** P < 0.01 and

*** P < 0.001 vs. tertile 1;

† *P < 0.05*,

‡ *P < 0.01 vs. tertile 2*.

Table 3 summarizes univariate correlates of MSNA and NC. In men, NC and insulin AUC_0−120_ were the strongest positive correlates of MSNA. In women, age and plasma LDL-cholesterol concentration were most closely associated with MSNA. In adjusted stepwise regression analyses, NC alone explained 12% of the variance in MSNA in men. Together with insulin AUC_0−120_, NC explained 22% of the variability in men (Table 4). In women, burst frequency was independently associated with age and HOMA-IR, which explained 19% of the variance, whilst burst incidence was independently associated with LDL-cholesterol and HOMA-IR, together explaining 18% of the variance (*P* < 0.05).

**Table 3:** **Univariate correlates of neck circumference and muscle sympathetic nerve activity (MSNA) within each gender**.

**Variables**	**Men (*****n*** = **72)**			**Women (*****n*** = **53)**		
	**Neck circumference (cm)**	**MSNA (bursts/min)**	**MSNA (bursts/100 hb)**	**Neck circumference (cm)**	**MSNA (bursts/min)**	**MSNA (bursts/100 hb)**
Neck circumference (cm)		0.35^**^	0.26^*^			
MSNA (bursts/min)	0.35^**^					
MSNA (bursts/100 hb)	0.26^*^					
Age (years)					0.33^*^	0.24
Body weight (kg)	0.73^***^			0.74^***^		
Body mass index (kg/m^2^)	0.74^***^	0.24^*^	0.21	0.61^***^		
Waist circumference (cm)	0.73^***^			0.70^***^		
Waist: Hip ratio	0.26^*^			0.50^***^		
Log leptin (ng/ml)	0.34^**^					
LDL-cholesterol (mmol/L)					0.28^*^	0.32^*^
Log triglycerides (mmol/L)	0.23^*^					
NEFA (mEq/L)	0.21	0.26^*^				
Log hs-CRP (mg/L)				0.26		
Log ALT (U/L)	0.21	0.31^**^				
Fasting insulin (mU/L)	0.31^**^					
HOMA-IR	0.30^*^				0.23	0.26
Log Matsuda ISI	−0.32^**^	−0.26^*^				
Insulin AUC_0−120_ (mU min L^−1^)	0.23	0.35^**^				
Diastolic BP (mmHg)		0.20				
Heart rate (bpm)	0.26^*^	0.43^***^			0.44^***^	

*Pearson correlation coefficients with P < 0.10 have been included in the table*.

* *P ≤ 0.05*,

** P ≤ 0.01, and

*** *P ≤ 0.001*.

**Table 4:** **Stepwise regression analyses of muscle sympathetic nerve activity (MSNA) in men (***n*** = 72)**.

**Model**	**Predictor variables**	**Standardized coefficients**	**Standard error**	***R*^2^**	***P***
***MSNA burst frequency (bursts/min)***					
1	Step 1 Neck circumference (cm)	0.348	0.38	0.12	0.003
2	Step 1 Neck circumference (cm)	0.295	0.38	0.12	0.01
	Step 2 Log ALT (U/L)	0.249	6.13	0.18	0.03
3	Step 1 Neck circumference (cm)	0.295	0.38	0.12	0.01
	Step 2 Log ALT (U/L)	0.249	6.13	0.18	0.03
4	Step 1 Neck circumference (cm)	0.312	0.39	0.14	0.006
	Step 2 Insulin AUC_0−120_ (mU min L^−1^)	0.279	0.00	0.22	0.02
***MSNA burst incidence (bursts/100 hb)***					
1	Step 1 Neck circumference (cm)	0.262	0.57	0.07	0.03
2	Step 1 Neck circumference (cm)	0.262	0.57	0.07	0.03
3	Step 1 Neck circumference (cm)	0.262	0.57	0.07	0.03
4	Step 1 Neck circumference (cm)	0.262	0.57	0.07	0.03

*Model 1. Variables entered: neck circumference (cm), age (years)*.

*Model 2. Variables entered: Model 1 and adiposity indices- BMI (kg/m^2^), waist circumference (cm), log leptin (ng/ml), log ALT (U/L)*.

*Model 3. Variables entered: Model 2 and lipid and blood pressure indices- LDL-C (mmol/L), log HDL-C (mmol/L), log triglycerides (mmol/L), NEFA (mEq/L), systolic blood pressure*.

*Model 4. Variables entered: Model 3 and indices of hyperinsulinemia and insulin resistance- log Matsuda ISI, insulin AUC_0−120_ (mU min L^−1^)*.

## Discussion

The present study investigated the association of NC, a potential marker for upper body fat deposition, with resting sympathetic neural activity in un-medicated middle-aged persons who were overweight or obese. Findings indicated a dichotomy of effects within the two genders. In men, NC was positively associated with MSNA independent of age, blood pressure and other adiposity measures such as waist circumference and BMI. In women, NC was associated with anthropometric measures, but did not delineate MSNA, metabolic or cardiovascular risk factors. Our data suggest that hyperinsulinemia secondary to decreased insulin sensitivity, gauged as elevated fasting and post-glucose insulin AUC_0−120_, may be an intermediary mechanism underlying the association between NC and MSNA in men. NC was not associated with spontaneous cardiac BRS in either gender in the present study.

Our findings concur with previously published data in Caucasian and Asian populations, showing an association of NC with classic obesity indices such as waist circumference and BMI, and with insulin-resistance related factors (Laakso et al., 2002; Zhou et al., 2013). More recent imaging studies have highlighted that NC is positively correlated with visceral adipose tissue and epicardial fat (Li et al., 2014; Küç¨uk et al., 2016) and with subclinical and clinical atherosclerosis (Zen et al., 2012; Baena et al., 2016). Our data further extend these observations, by demonstrating for the first time that NC is correlated with MSNA, a direct measure of central sympathetic neural outflow to skeletal muscle, in men. Various pathogenic mechanisms may be responsible for this association. Firstly, hyperinsulinemia, which related to both NC and MSNA, is known to elicit sympathetic activation via central and peripheral mechanisms (Anderson et al., 1991; Kern et al., 2005). Centrally, insulin's effects are initiated in the arcuate nucleus and involve various neural signaling pathways that mediate organ-specific sympathetic outflows (Rahmouni et al., 2004; Cassaglia et al., 2011). Peripherally, insulin may evoke a reflex increase in SNS activity in response to its vasodilatory effects, albeit insulin-induced sympathetic activation and vasodilation in skeletal muscle are known to be blunted in obese humans (Vollenweider et al., 1994). Reverse causality may also be active, in that chronic sympathetic activation *per se* may worsen insulin resistance via hemodynamic and lipolytic effects, thus maintaining a vicious bidirectional cycle (Jamerson et al., 1993; Masuo et al., 1997). Interestingly, increasing adiposity was not associated with worsening insulin sensitivity in the women participants of our study.

Secondly, greater NC is associated with OSA syndromes (Karimi et al., 2010; Lim et al., 2014) which are known to be an important mediator of elevated sympathetic drive in obesity. This is attributed to the stimulation of peripheral chemoreceptors by the anoxia induced by nocturnal hypopneic-apnoeic episodes, and also to diminished arterial baroreflex sensitivity in OSA subjects (Narkiewicz et al., 1998b; Toschi-Dias et al., 2013). The prevalence of OSA in newly diagnosed metabolic syndrome subjects with similar clinical profile to our cohort, has been reported at 60% (Drager et al., 2010). In the absence of formal polysomnographic testing, we can only speculate about the potential involvement of OSA in the association between NC and MSNA in the present study. Notably, NC is more strongly associated with OSA in men than women, which may be one potential explanation for the gender differences observed in our study (Lim et al., 2014).

Whilst no study to date has examined the relation of NC to baroreflex function, it is recognized that visceral obesity and the metabolic syndrome are associated with impairment in baroreceptor ability to control heart rate and efferent postganglionic sympathetic nerve traffic (Beske et al., 2002; Grassi et al., 2005). Attenuation of these reflex mechanisms stemming from carotid and aortic baroreceptors is thought to play a causal role in the hyperadrenergic state typical of the metabolic syndrome. In the present study we found no relation between NC and spontaneous cardiac BRS, which primarily reflects vagal efferent activity, in either men or women. However, it merits emphasis that we did not analyse baroreceptor modulation of MSNA, which may be of particular relevance. Notably, in patients with OSA, impairment of baroreflex sympathetic modulation is not accompanied by any impairment in baroreflex control of heart rate (Narkiewicz et al., 1998b).

Dissimilarities in neck fat accumulation have been demonstrated in men and women using positron emission and computed tomography imaging. Overweight and obese women have significantly higher neck subcutaneous fat and significantly lower neck intermuscular adipose tissue compared to age- and BMI-matched men (Torriani et al., 2014). Posterior intermuscular and subcutaneous neck adipose tissue depots have been linked with cardiometabolic risk (Torriani et al., 2014). Moreover, NC is more closely related to abdominal visceral fat in men than women (Li et al., 2014). A number of previous studies, including our own work, support the notion that gender influences the interaction between adiposity indices and SNS activity. BMI and waist circumference are closely associated with sympathetic nerve traffic in men but not women (Lambert et al., 2007; Tank et al., 2008). The biological basis for these differences is yet to be elucidated (Brooks et al., 2015). There are recognized sex differences in the distribution, morphology and activity of adipose tissue. Adipose tissue generates various signals that may modulate sympathetic activity, including leptin, angiotensinogen and NEFA, and there may be gender-specific differences in the quantities produced and actions elicited on sympathetic pathways (Brooks et al., 2015). For example, rodent studies show that leptin's sympathoexcitatory effects are exaggerated in obese male animals, whereas in ovariactomized female rats, intracerebroventricular leptin failed to alter lumbar sympathetic nerve activity, unless the rats were given estrogen replacement therapy (Shi and Brooks, 2015). In diet-induced obese mice, weight gain was associated with increased plasma angiotensin II in male, but not female animals (Gupte et al., 2012). Blunted leptin-mediated sympathetic activation, quantified as whole-body norepinephrine spillover rate, has also been linked to leptin receptor polymorphisms, highlighting that genetic factors may modulate the relation between adiposity hormones and SNS activity (Masuo et al., 2008). As far as the present study is concerned, we found positive associations between NEFA, MSNA and NC, and leptin and NC in the men, but no further relationships with other adiposity signals. Insulin resistance is the primary mechanisms leading to greater free fatty acid release in upper body obesity. Enhanced lipolysis produced by activation of the SNS is another contributing factor. In turn, NEFA promote insulin resistance via the Randle cycle and may thus increase SNS activity indirectly (via hyperinsulinemia). Moreover, lipid administration studies suggest that NEFA may also have direct effects on α1-adrenoceptor sensitivity and central sympathetic drive (Stepniakowski et al., 1995; Florian and Pawelczyk, 2010).

The strengths of our study include the use of un-medicated subjects to avoid confounding by pharmacological agents. Furthermore, microneurographic recordings are highly reproducible within individuals and provide a direct measure of postganglionic efferent nerve discharges leading to skeletal muscle vasculature, which are of particular relevance to vascular tone and glucose utilization (Fagius and Wallin, 1993; Jamerson et al., 1993; Fairfax et al., 2013; Gamboa et al., 2014). Our study also has several limitations. First, NC is a measure that comprises both adipose and lean tissue and our study does not distinguish between these compartments. Second, the sample size was relatively small and we did not include any lean controls, which would have enhanced the heterogeneity of the cohort, and possibly the strength of associations. Third, a number of unmeasured factors such as OSA, fitness level and androgenicity may have influenced the relationships that were studied. Fourth, we only examined sympathetic outflow to skeletal muscle but not to other organs such as the kidneys, which are of relevance to the development of hypertension and cardiovascular risk. Finally, the cross-sectional design does not permit insight into causality.

In summary, within overweight and obese men NC was associated with MSNA and features of insulin resistance independent of central adiposity. In women NC was associated with anthropometric measures but not with MSNA or metabolic indices. Given the clinical relevance of chronic sympathetic overactivity to future cardiovascular and metabolic disease (Masuo et al., 1997; Schlaich et al., 2015; Wulsin et al., 2015), NC may be a simple measure to aid risk stratification in at risk overweight and obese men. Our data does not support its use in women, beyond identifying overweight and obesity.

## Author contributions

NS and EL conceived the study. NS, MG, CS, PN, and MS collected clinical data. EL performed microneurographic recordings and analyses. NE and SP performed laboratory assays. NS performed statistical analyses, wrote and revised the manuscript. NS, PN, JD, GL, and EL interpreted the data. All authors critically read and approved the manuscript.

## Funding

This study was funded by a Diabetes Australia Millennium Grant in type 2 diabetes to NS. JD, GL, and MS are supported by NHMRC Fellowships. We also wish to acknowledge the Victorian Government's Operational Infrastructure Support Program.

### Conflict of interest statement

PN has consultative and advisory board associations with Merck Sharp & Dohme and Astra Zeneca. JD is a consultant for Apollo Endosurgical, Bariatric Advantage, and is a member of the Optifast^®^ Medical Advisory Board for Nestle Health, Australia. GL has acted as a consultant for Medtronic and has received honoraria from Medtronic, Pfizer and Wyeth Pharmaceuticals for presentations. MS serves on scientific advisory boards for Abbott Pharmaceuticals, Novartis Pharmaceuticals and Medtronic and has received research support and travel support, lecture fees and honoraria from Abbott, Novartis, Servier, Boehringer Ingleheim, and Medtronic. These organizations played no role in the design, analysis or interpretation of data described here, nor in the preparation, review, or approval of the manuscript. The other authors declare that the research was conducted in the absence of any commercial or financial relationships that could be construed as a potential conflict of interest.
